# Optic disc hemorrhage in a young female following mRNA coronavirus disease 2019 vaccination: a case report

**DOI:** 10.1186/s13256-022-03690-3

**Published:** 2022-12-08

**Authors:** Kyohei Tsuda, Akio Oishi, Takashi Kitaoka

**Affiliations:** Department of Ophthalmology and Visual Sciences, Nagasaki University, 1-7-1, Sakamoto, Nagasaki, 852-8501 Japan

**Keywords:** COVID-19 vaccine, Optic disc hemorrhage, Retinal hemorrhage, Adverse event

## Abstract

**Background:**

Since the development of the coronavirus disease 2019 vaccine, there have been many reports of its adverse effects. While respiratory symptoms are common, many other symptoms in various organs have been reported. Herein, we report a case of optic disc and retinal hemorrhage that developed immediately after coronavirus disease 2019 vaccination.

**Case presentation:**

A healthy 18-year-old Japanese female noticed floater in the left eye 1 day after the second vaccination for coronavirus disease 2019 (Pfizer Inc.). Her visual acuity was 20/20 in the left eye, and Goldmann visual field test showed a relative scotoma around blind spot and in the temporal lower quadrant. It was considered due to subretinal hemorrhage and optic disc swelling. Fundus examination revealed retinal and optic disc hemorrhage. Pupillary reflex was intact and central critical flicker was not impaired, indicating that optic nerve was not involved. There was no sign of inflammation, vascular abnormality, nor history of an intense Valsalva maneuver. The hemorrhage resolved spontaneously within 5 months.

**Conclusion:**

This case expands the clinical presentation of coronavirus disease 2019 vaccination-associated ocular adverse events, and it should be kept in mind when patients with similar symptoms visit clinics. The case report will help clinicians avoid unnecessary and invasive examinations and treatment.

## Background

In response to the worldwide coronavirus disease 2019 (COVID-19) pandemic, an messenger ribonucleic acid (mRNA) vaccine was developed very rapidly. Although the vaccine is widely used and exhibits excellent efficacy [1], various vaccine-related adverse events have been reported. Common adverse events include fever, headache, muscle and joint pain, cough, wheezing, stridor, hoarseness, rhinorrhea, tachycardia, and weak/absent central pulse [2]. Serious events such as anaphylactic shock and myocarditis have also been rarely reported [2, 3]. Ocular adverse reactions such as eyelid swelling, herpetic keratitis, corneal graft rejection after corneal transplantation, acute macular neuroretinopathy, serous retinal detachment, uveitis, optic neuropathy, eye movement disorders, retinal vein occlusion, and acute retinal necrosis due to varicella zoster virus reactivation have been reported [4]. However, to the best of our knowledge, optic disc hemorrhage has not been reported to date after COVID-19 vaccination. Herein, we present the case of a female who developed optic disc hemorrhage after the mRNA COVID-19 vaccination.

## Case presentation

**Fig. 1:**
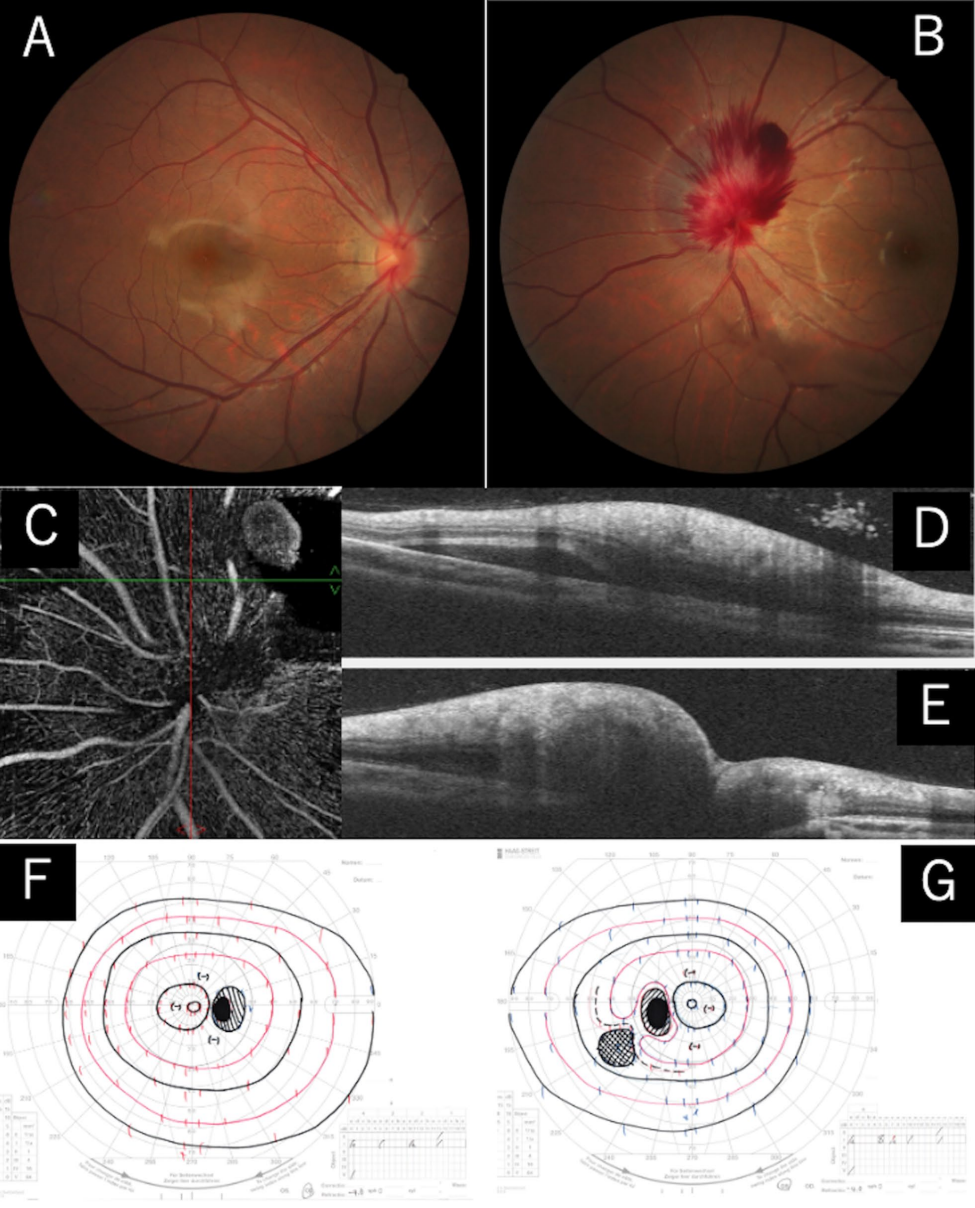
Clinical images of optic disc hemorrhage after COVID-19 vaccination. Fundus photography revealing a retinal hemorrhage around the left optic disc. Fundus photographs of the right eye showing no abnormal findings (**A**, **B**). Optical coherence tomography angiography revealing no abnormal vessels (**C**). Optical coherence tomography showing hemorrhage in the subretinal, intraretinal, and preretinal spaces in the left eye (**D** corresponds to the red line in **C**, and **E** corresponds to the green line). Kinetic perimetry of the left eye revealing a relative scotoma in the temporal lower quadrant. The right eye showing normal results (**F** and **G**)

An 18-year-old Japanese female presented with complaint of floaters in the left eye. She was myopic and was using glasses since elementary school but did not have any other ocular or systemic diseases. Ophthalmological examinations a few months earlier revealed no abnormal findings. She received the second COVID-19 vaccine (Pfizer Inc.) after the uneventful first vaccination and became aware of floaters in her left eye the following day. She visited an ophthalmology clinic 8 days later when her symptoms worsened. A retinal hemorrhage was observed around the optic disc, and the patient was referred to our department 10 days after the vaccination. Her blood pressure was 103/56 mmHg and her heart rate was 76 beats per minute. Her visual acuity was 20/20 and the intraocular pressure was 15 mmHg in both eyes. The critical flicker fusion frequency was 37.5 Hz and 37.0 Hz in the right and left eye, respectively. There was no family history, medication, preceding symptoms, nor an intense Valsalva maneuver. Ophthalmologic examination revealed no relative afferent pupillary defect and no sign of inflammation. Hemorrhage around the left optic disc and accompanying disc swelling was noted. The hemorrhage extended from the subretina to the preretina. A mild vitreous hemorrhage was also observed and was considered the cause of the floaters. The patient underwent fundus photography, kinetic visual fields on the Goldmann perimeter (GP), optical coherence tomography (OCT), and OCT angiography (OCTA); the images are shown in Fig. 1.

**Fig. 2:**
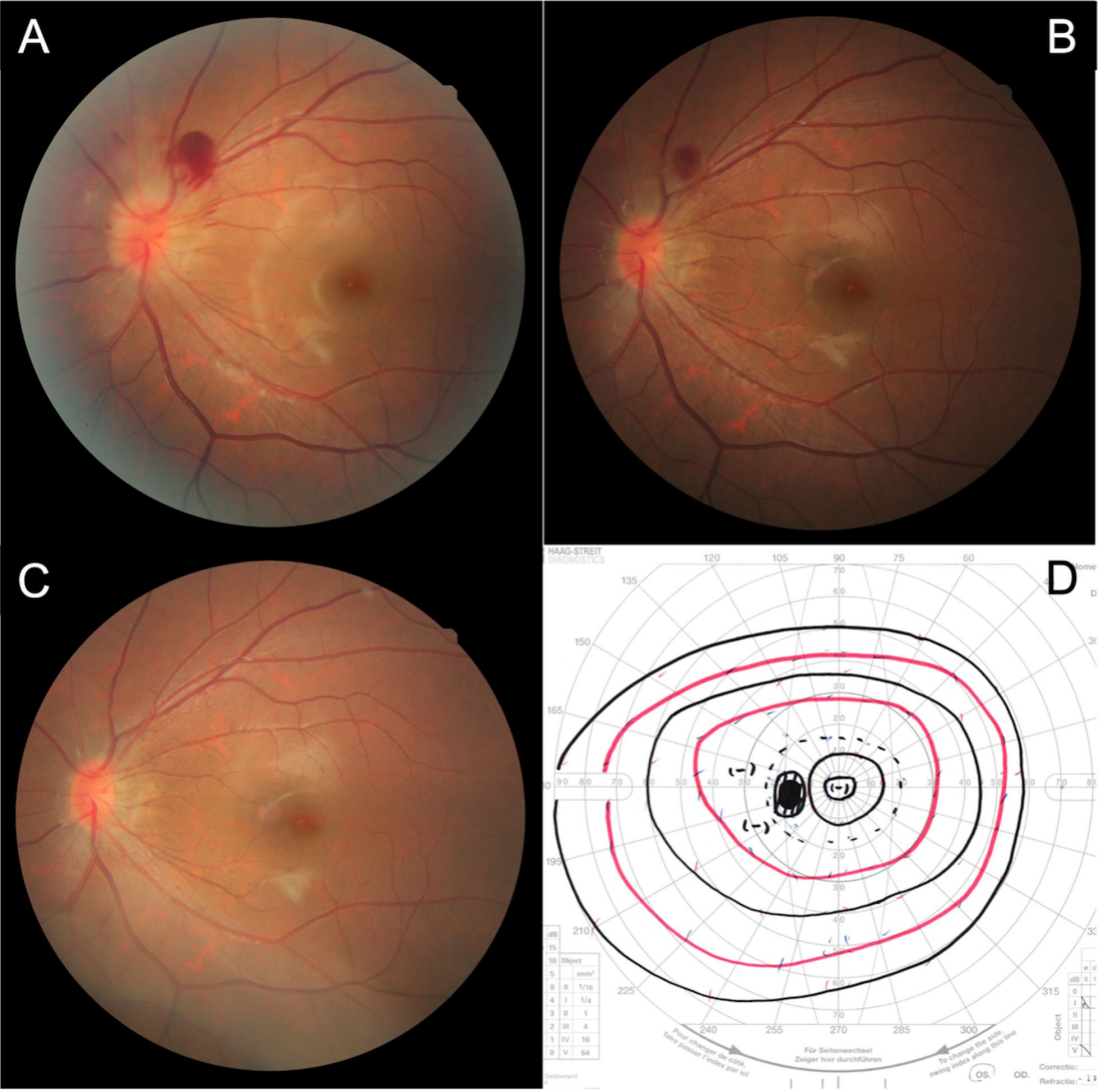
Fundus photographs taken 1 (**A**), 2 (**B**), and 5 (**C**) months later, showing that the hemorrhage and optic disc swelling gradually improved. Five months later, fundus photography and kinetic perimetry showed completely normal result (**C**, **D**)

We considered papilledema, optic neuritis, ischemic optic neuropathy, optic neuroretinitis, Terson syndrome, posterior vitreous detachment, vascular tumor, uveitis, and Valsalva retinopathy as the differential diagnoses. Papilledema and Terson syndrome were considered unlikely because the findings were monocular, and the patient did not have headache, neurologic signs, or medical history. Optic neuritis, ischemic optic neuropathy, and optic neuroretinitis were also less likely because there were no visual defects, despite the prominent hemorrhage. Posterior vitreous detachment was absent and hence was not the cause of the hemorrhage. Uveitic entities such as sarcoidosis were not likely because there was no inflammatory findings in the anterior chamber and vitreous cavity. Valsalva retinopathy was suspected but there was no history of an intense Valsalva maneuver and the finding was not very typical. We kept her under observation without any medication. The findings gradually regressed within 5 months (shown in Fig. 2).

## Discussion and conclusions

We reported a case with optic disc hemorrhage that developed following mRNA COVID-19 vaccination. Though the pathogenesis is unclear, the timing of the event (within 1 day of vaccine) is highly suggestive of a cause-and-effect relationship.

A previous study investigated retinal hemorrhage following the COVID-19 vaccination [5]. They reported that 12 patients developed age-related macular degeneration-related submacular hemorrhage, and 11 developed retinal vein occlusion in median of 2 days after vaccination. They discussed that patients who originally have microvascular disease are more likely to develop hemorrhagic complications. The spike protein itself or immune response to the protein are considered to cause platelet activation, vein occlusion, and retinal hemorrhage [5]. In our case, patient was young and had no systemic diseases, and the hemorrhage was not associated with vein occlusion nor macular degeneration. The mechanism of the hemorrhage in the present case would be different from these cases.

Although the finding was not typical and there was no evident episode of Valsalva maneuver, we cannot reject the possibility of Valsalva retinopathy. Valsalva retinopathy is a disorder in which venous blood cannot return to the thoracic cavity due to a sudden increase in intrapleural pressure caused by holding one’s breath (Valsalva effect), resulting in increased venous pressure, retinal vessel rupture, and hemorrhage [6, 7]. The hemorrhage is usually below the internal limiting membrane but may extend into the vitreous space. Indeed, preretinal hemorrhage and mild vitreous hemorrhage were noted in the present case. The causes include muscle training, coughing, vomiting, labor, sexual activity, bungee jumping, excessive straining, and heavy lifting [7], but sometimes it develops with no obvious Valsalva effect [8]. The prognosis of Valsalva retinopathy is generally good without treatment [6]. Our case also showed spontaneous resolution without any treatment. As to the COVID-19 infection, there is a case report that a patient developed Valsalva retinopathy due to coughing [9]. Meanwhile, the present case did not have coughing.

Alternatively, considering that cardiovascular events such as myocarditis and thrombosis are known to occur after COVID-19 vaccination [3, 4], there might be temporal changes such as vasospasm in circulatory dynamics. The optic disc was relatively small, and it is known that such small crowded discs are risk factors for ischemic optic neuropathy because vessels and nerve fibers have to pass through small scleral canal openings and are susceptible to circulatory disturbance. The small disc might have contributed to the development of hemorrhage in this case despite the presentation not being ischemic optic neuropathy. We also considered optic disc drusen, but it was denied based on OCT and fundus autofluorescence. Otherwise, it can be just a coincidental co-occurrence. In any event, further studies are required to study this association.

In summary, we reported a case of optic disc hemorrhage after the mRNA COVID-19 vaccination. This report expands the catalogue of mRNA COVID-19 vaccine-associated ocular adverse events and helps clinicians avoid unnecessary and invasive examinations and treatments.

## Data Availability

Data sharing is not applicable to this article as no datasets were generated or analyzed during the current study.

## Abbreviations

COVID-19: Coronavirus disease 2019
GP: Goldmann perimeter
OCT: Optical coherence tomography
OCTA: OCT angiography
